# Rare mutations in *Pfmdr1* gene of *Plasmodium falciparum* detected in clinical isolates from patients treated with anti-malarial drug in Nigeria

**DOI:** 10.1186/s12936-019-2947-z

**Published:** 2019-09-18

**Authors:** Abel O. Idowu, Wellington A. Oyibo, Sanjib Bhattacharyya, Manjeet Khubbar, Udoma E. Mendie, Violet V. Bumah, Carolyn Black, Joseph Igietseme, Anthony A. Azenabor

**Affiliations:** 1 Department of Biomedical Sciences, College of Health Sciences, University of Wisconsin, 2400 E. Hartford Avenue, Milwaukee, WI 53211 USA; 2 Department of Pharmaceutics and Pharmaceutical Technology, Faculty of Pharmacy, University of Lagos, Lagos, Nigeria; 3 City of Milwaukee Health Department Laboratory, Milwaukee, USA; 4 ANDI Centre of Excellence in Malaria Diagnosis, College of Medicine, University of Lagos, Lagos, Nigeria; 5 Molecular Pathogenesis Laboratory, National Center for Emerging and Zoonotic Infectious Diseases, Centers for Disease Control and Prevention, Atlanta, GA USA; 6 Department of Biology, North Life Science 317, San Diego State University, San Diego, CA 92182 USA

**Keywords:** Malaria, Infectious disease epidemiology, Antimalarial gene polymorphism, Parasitic disease epidemiology and control, Plasmodium

## Abstract

**Background:**

*Plasmodium falciparum*, the deadliest causative agent of malaria, has high prevalence in Nigeria. Drug resistance causing failure of previously effective drugs has compromised anti-malarial treatment. On this basis, there is need for a proactive surveillance for resistance markers to the currently recommended artemisinin-based combination therapy (ACT), for early detection of resistance before it become widespread.

**Methods:**

This study assessed anti-malarial resistance genes polymorphism in patients with uncomplicated *P. falciparum* malaria in Lagos, Nigeria. Sanger and Next Generation Sequencing (NGS) methods were used to screen for mutations in thirty-seven malaria positive blood samples targeting the *P. falciparum* chloroquine-resistance transporter (*Pfcrt*), *P. falciparum* multidrug-resistance 1 (*Pfmdr1*), and *P. falciparum kelch 13* (*Pfk13*) genes, which have been previously associated with anti-malarial resistance.

**Results:**

Expectedly, the NGS method was more proficient, detecting six *Pfmdr1,* seven *Pfcrt* and three *Pfk13* mutations in the studied clinical isolates from Nigeria, a malaria endemic area. These mutations included rare *Pfmdr1* mutations, N504K, N649D, F938Y and S967N, which were previously unreported. In addition, there was moderate prevalence of the K76T mutation (34.6%) associated with chloroquine and amodiaquine resistance, and high prevalence of the N86 wild type allele (92.3%) associated with lumefantrine resistance.

**Conclusion:**

Widespread circulation of mutations associated with resistance to current anti-malarial drugs could potentially limit effective malaria therapy in endemic populations.

## Background

*Plasmodium falciparum* causes the highest number of malaria-related fatalities globally and has high prevalence in Nigeria [1, 2]. An estimated 212 million cases and 429,000 deaths due to malaria occurred in 2015, with 90% of the cases recorded in Africa [3]. Emergence and spread of mutations conferring drug resistance has limited the use of two affordable and previously effective anti-malarial drugs, chloroquine and sulfadoxine/pyrimethamine, leading to the recommendation by the World Health Organization (WHO) of artemisinin-based combination therapy (ACT), as the first-line treatment for malaria [4].

Artemisinin-based combinations are made up of a rapid but short-acting artemisinin and a long-acting partner drug combined to reduce the emergence of resistance. Artemether–lumefantrine (AL) and artesunate-amodiaquine are the recommended artemisinin-based combinations for the treatment of uncomplicated malaria in Nigeria, and their suitability has been confirmed by results of recent therapeutic efficacy studies (TES), which showed a cure rate of greater than 95% [5]. Deployment of ACT has led to a significant reduction in the number of malaria cases and fatalities [3], however, resistance to artemisinin, manifesting as delayed parasite clearance, has been observed in Western Cambodia since 2008 and has more recently been documented in extended areas of Southeast Asia [6–9]. The fear that artemisinin resistance could also spread through a similar trajectory as was observed with chloroquine and sulfadoxine/pyrimethamine resistance from Southeast Asia to Africa is now a real concern.

Apart from TES, which is a gold standard to monitor anti-malarial therapeutic efficacy, the WHO recommends the use of molecular markers to monitor the emergence of mutations associated with resistance to anti-malarial drugs as a proactive exercise to detect emerging resistance and prevent potential future treatment failure [10]. Several genetic markers associated with resistance to anti-malarial drugs have been identified. Mutations in the kelch propeller domain of the *k13* gene have been identified as molecular markers for artemisinin resistance by evaluating in vitro survival assays and observing delayed parasite clearance times and are currently being used to track the spread of resistance [7]. Currently, five validated (by in vivo and in vitro correlation data) and eight candidate (by correlation with delayed parasite clearance) *k13* mutations have been associated with artemisinin resistance [11]. Polymorphisms in the *P. falciparum* chloroquine resistance transporter gene (*Pfcrt*) with substitutions at codon positions 72 to 76, 97, 220, 271, 326, 356, and 371 have been associated with in vivo and in vitro resistance to chloroquine and amodiaquine [12, 13]. The substitution at codon 76 (K76T) is the most predictive point mutation for chloroquine resistance [13].

The *Pfmdr1* amino acid mutations that have been implicated in multidrug resistant phenotypes include the amino-terminal mutations (N86Y and Y184F) and the three carboxyl-terminal mutations (S1034C, N1042D and D1246Y) on the *Pfmdr1* gene. These mutations have been reported to increase resistance to chloroquine, as well as modulate malaria parasite sensitivities to multiple drugs such as mefloquine, amodiaquine, quinine and halofantrine [14–16]. Mutations in *Pfmdr1* is further suspected to be involved in parasite susceptibility to each of the ACT partner drugs as well as trophozoite-stage parasite sensitivity to artemisinin derivatives [17–19].

Recent studies have evaluated the emergence of resistance to piperaquine, an artemisinin partner drug [20, 21], with one study confirming that 14 out of 40 clinical isolates exhibited piperaquine resistance, leading to the recommendation for a change of treatment in Vietnam [22]. To date, no molecular marker for piperaquine resistance has been identified [10], although a recent study has linked a surrogate marker to piperaquine resistance [23], highlighting the need for the use of molecular studies (involving whole genome sequencing) as a realistic approach to detect early emergence of resistant populations of parasites in areas where ACT is used. In select studies, low levels of *k13* mutations were detected in parasite isolates obtained from various regions of Africa [8, 24, 25]. In Nigeria, although chloroquine was replaced by artemisinin-based combinations as the drug of choice, a recent study observed that the percentage of children with malaria treated with un-recommended anti-malarial monotherapy of chloroquine and sulfadoxine/pyrimethamine was still significantly high at 11.6% and 10%, respectively, compared to ACT at 7.5% [26]. As sometimes observed, heightened use of ACT and other anti-malarial therapies may confer transmission advantage to drug resistant *P. falciparum*. Consequently, self-medicating and indiscriminate drug use may influence the circulating population of parasite strains, thus, selecting for mutant parasites through selective pressure. There is a need for continuous monitoring for the emergence of new anti-malarial-associated mutations that may arise in these high-endemic and highly populated areas, such as Nigeria.

This study, using Sanger and NGS methods, reported here, reveal the mutations identified in the *Pfcrt, Pfmdr1* and *Pfk13* genes and highlight the detection of rare mutations in the *Pfmdr1* gene in Nigeria.

## Methods

### Study sites

The study participants were drawn from two sites in Lagos State; Ikorodu and Amukoko. Investigators from the University of Lagos have been conducting routine surveillance for malaria in Ikorodu, a semi urban area and Amukoko an urban slum in Lagos state. The two sites are hyper-endemic for *P. falciparum*, with generally high malaria transmission and a seasonal peak from July to November. Samples were collected between July 2015 and July 2016. The samples from Amukoko were collected at the St. Matthew Catholic Hospital. In Ikorodu, located approximately 20 km from Lagos, study participants were enrolled at primary health centres, Ijede and Agura.

### Study samples

A cross section sample of research participants who were patients, who reported to these health care facilities, with history of headache, fever (axillary temperature ≥ 37.5 °C) and other clinical symptoms of malaria in the previous 24 h, was screened for malaria infection by both malaria rapid diagnostic test (RDT) (SD Bioline-Pf/PAN) and microscopy. Recruitment was on a voluntary basis with informed consent obtained from all participating patients/caregivers after due counseling. The consent forms and all information about the study participants were kept confidential. A questionnaire was administered to each research participant to collect information such as demographic data, history of fever, other symptoms, and medication history, and a case-controlled design was adopted. Healthy individuals who are staff and students at the College of Medicine, University of Lagos that were without symptoms of acute illness during screening, had no evidence or history of chronic illness, and had no parasitaemia upon examination were also enrolled as controls. Venous blood collected into EDTA container from each research participant was used for malaria diagnosis and spotted on Whatman^®^ #3 filter paper for molecular studies.

Patients were treated with a combination of artemether and lumefantrine (AL [Coartem^®^]), as recommended by the WHO and the Nigeria National Malaria Control Programme (NMCP). Samples from subjects aged 2 to 73 years with uncomplicated *P. falciparum* mono-infection confirmed by microscopy (parasite density of 2000 to 199,999 asexual parasites/μL) were used for further study.

### Sanger sequencing

Genomic DNA was isolated from three punched-out circles from a dried blood spot or 50 μl of whole blood taken at enrollment (day 0) using a QIAamp DNA minikit (Qiagen, Valencia, CA) according to the manufacturer’s protocol.

Confirmation of *P. falciparum* infection and DNA quality assessment were conducted using photo-induced electron transfer (PET)-PCR [27]. The confirmed *P. falciparum* samples were used to amplify the *Pfk13* propeller domain, *Pfmdr1* and *Pfcrt* genes using previously described methods [28, 29]. Briefly, PCR was performed to amplify the full-length *Pfcrt, Pfmdr1* and *Pfk13* genes using the New England BioLabs (NEB) High Fidelity PCR kit (New England BioLabs, USA; catalog no. 51104) according to the manufacturer’s instructions with a 20 μL master mix preparation using the 5 μL GC buffer and 1–2 μL of total genomic DNA. PCR products were confirmed after ExoSAP cleanup using 1.5% agarose gel electrophoresis. The samples were then genotyped by direct sequencing using an Applied Biosystems 3130 capillary sequencer and analysed with Geneious Pro R7 (Biomatters, Inc., Auckland, New Zealand) to identify specific SNPs. Sequencing and data analysis was performed at the Centers for Disease Control and Prevention, Atlanta, GA.

### Next Generation Sequencing (NGS)

The recently developed MaRS protocol was employed as previously described [30]. Briefly, a Sequal Prep kit (ThermoFisher Scientific; catalog number A1051001) was used to purify and normalize all the PCR products to 1.0 to 2.0 ng/μL and 10.0 μL of each PCR product was then pooled for each sample. Illumina-supported sequencing adaptors and unique sequence indices were added to the pooled PCR amplicons using the Illumina Nextera XT kit (Illumina; catalog numbers FC-131-1096 and FC-1311002) to generate a unique sequence barcode identifier (ID) for each sample and all pooled gene targets. Next, a limited-cycle PCR was performed to amplify the fragmented DNA and add unique (sequence) indices to each pooled sample. PCR products were then size selected (< 300 bp), normalized, and purified using Agent court AM pure XP beads (Beckman Coulter Genomics; catalog number A63881). Following normalization, 5.0 μL of each library was pooled, allowing up to 384 libraries (or individual samples) to be pooled on a single Illumina MiSeq run. For data quality control of the FASTQ files obtained, only Q30 scores of > 98% were used. Low phred scores sequences were not considered in the analysis. The reference used to call the SNP and the haplotypes was the *P. falciparum* 3D7. The bioinformatics analysis of the data was performed using the Geneious R10 software package. The threshold used for SNP calling was set at allele frequency of > 10%.

## Results

### Detection of *Pfk13, Pfcrt*, and*Pfmdr1* mutations by Sanger and NGS methods

Clinical samples from 1500 patients in Ikorodu and 730 in Amukoko study sites which were screened for malaria by rapid diagnostic test (RDT) and microscopy yielded 98 malaria-positive samples that were evaluated further by PET-PCR methods.

Thirty-seven samples from patients with self-reported anti-malarial medication use that were positive for *P. falciparum* were selected for molecular studies. Amplification and sequencing of the *Pfk13, Pfcrt,* and *Pfmdr1* genes was successfully performed in 26 samples that passed the quality threshold based on a minimum CT value of 40; the rest samples had low parasitaemia and amplification was not successful. In order to determine the efficiency of using the Sanger and NGS methods to detect mutations in the clinical isolates, both methods were used for sequencing of the selected genes. The Sanger method was limited to sequencing of partial fragments within the genes, however, the NGS method allowed for the sequencing of the entire genes. Of the common fragments sequenced by both methods, more mutations and haplotypes were detected by NGS than by Sanger. Furthermore, additional mutations and haplotypes were identified in the regions that could only be sequenced by the NGS method, thus, the NGS method has shown to be a more sensitive detection method for the screening of mutations in these three genes (Tables 1, 2). These results indicate the preeminence of NGS for use in the tracking of the emergence and spread of mutant alleles.

**Table 1:** Distribution of mutant and wild type alleles in the samples sequenced by Sanger and NGS methods

Gene	Mutations	Sanger number (%)		NGS number (%)	
		Mutant allele	Wild type	Mutant allele	Wild type
*Pfcrt*	M74I	7 (26.9%)	19 (73.1%)	9 (34.6%)	17 (65.4%)
	N75E	7 (26.9%)	19 (73.1%)	9 (34.6%)	17 (65.4%)
	K76T	7 (26.9%)	19 (73.1%)	9 (34.6%)	17 (65.4%)
	A220S	NA	NA	7 (26.9%)	19 (73.1%)
	Q271E	NA	NA	6 (23%)	20 (77%)
	I356T	NA	NA	8 (30.8%)	18 (69.2%)
	R371I	NA	NA	7 (26.9%)	19 (73.1%)
*Pfmdr1*	N86Y	2 (7.7%)	24 (92.3%)	2 (7.7%)	24 (92.3%)
	Y184F	17 (65.4%)	9 (34.6%)	22 (84.6%)	4 (15.4%)
	N504K	NA	NA	4 (15.4%)	22 (84.6%)
	N649D	NA	NA	3 (11.5%)	23 (88.5%)
	F938Y	NA	NA	3 (11.5%)	23 (88.5%)
	S967N	NA	NA	1 (3.8%)	25 (96.2%)
*Pfk13*	Q613H	Nil	100%	1 (3.8%)	25 (96.2%)
	K189T	NA	NA	17 (65.4%)	9 (34.6%)
	H136N	NA	NA	1 (3.8%)	25 (96.2%)

The mutations detected in the *Pfcrt, Pfmdr1,* and *Pfk13* genes that were sequenced by Sanger and NGS methods are displayed. The total number of samples that carried the specific mutation is shown and percent is given in parentheses. NA, not applicable. Unlike NGS, Sanger could only sequence fragments and not full length of the genes

**Table 2:** Distribution of the haplotypes identified in the samples sequenced by Sanger and NGS methods

Gene	Haplotypes	Sanger (number %)	NGS (number %)
*Pfcrt*	CVIET	7 (26.9%)	8 (30.8%)
	CVMET	0	1 (3.8%)
	CVMNK (wt)	19 (73.1%)	17 (65.4%)
	SEII	ND	1 (3.8%)
	SETI	ND	6 (23%)
	AEIR	ND	1 (3.8%)
	AQIR	ND	18 (69.2%)
*Pfmdr1*	YF	2 (7.7%)	2 (7.7%)
	NF	15 (57.7%)	22 (84.6%)
	NY (wt)	9 (34.6%)	2 (7.7%)
	KNYS	ND	1 (3.8%)
	KDFS	ND	2 (7.7%)
	NDFS	ND	1 (3.8%)
	KNFN	ND	1 (3.8%)
	NNYS	ND	2 (7.7%)
	NNFS (wt)	ND	19 (73.1%)

The haplotypes identified in the *Pfcrt* and *Pfmdr1* genes that were sequenced by Sanger and NGS methods are shown. Wt = wild type. The total number of samples that carried the specific haplotype is indicated and percent is given in parentheses. ND = not detected. The ability to detect certain haplotypes by Sanger is limited because only fragments and not full length gene could be sequenced by the method. The *Pfcrt* mutant haplotypes CVIET, CVMET and wild type CVMNK are based on protein sequence at codons 72–76 while *Pfcrt* mutant haplotypes SEII, SETI, AEIR and wild type AQIR are based on protein sequence at codons 220,271,356 and 371. The mutant *Pfmdr1* haplotypes YF, NF and wild type NY are based on protein sequence at codons 86 and 184 while mutant *Pfmdr1* haplotypes KNYS, KDFS, NDFS, KNFN, NNYS and wild type NNFS are based on protein sequence at codons 504,649,938 and 967

### Prevalence of mutations detected in the *Pfk13, Pfcrt*, and *Pfmdr1* genes

Since more mutations were detected by NGS than by Sanger, it was necessary to investigate the prevalence of mutations in the each of the *Pfcrt, Pfmdr1,* and *Pfk13* genes because some specific mutations in those genes have been shown to have implications for anti-malarial resistance. Mutations were detected in 73.1% of studied samples in the *Pfk13* gene. Investigations of the common regions of the *Pfk13* gene sequenced by both methods showed that the NGS method detected a propeller mutation (Q613H), which was undetected by the Sanger method. An additional two non-propeller mutations (K189T, H136N) in the *Pfk13* gene were detected in the additional genomic regions sequenced by NGS only; see Table 1. When evaluating the genomic regions sequenced by both Sanger and NGS methods, the mutations detected in the *Pfcrt* gene, the M74I, N75E and K76T mutations were found in only 7 (26.9%) of the samples by Sanger and 9 (34.6%) of the samples by NGS. The CVIET and CVMET haplotypes detected in the regions sequenced by both methods were found in one additional sample by NGS than by Sanger. Furthermore, four additional mutations (A220S, Q271E, I356T, and R371I) and three additional mutant haplotypes (SEII, SETI, AEIR) were identified in additional regions of the *Pfcrt* gene sequenced by NGS only; see Tables 1, 2. This result revealed that approximately one-third of the samples analysed had one or more *Pfcrt* mutations, including a key mutation, K76T, which has been previously associated with resistance to the formerly used chloroquine and the current alternate first-line ACT partner drug amodiaquine.

Evaluation of the mutations detected in the common regions of the *Pfmdr1* gene sequenced by Sanger and NGS methods, revealed that the Y184F mutation and the NF haplotype were found in five additional samples by NGS compared to Sanger. In the regions sequenced by NGS only, an additional four mutations (N504K, N649D, S967N, F938Y) and 5 mutant haplotypes (KDFS, NDFS, KNFN, KNYS, NNYS) were detected; see Tables 1, 2. The S967N, N649D, F938Y, and N504K mutations that were detected in 1, 3, 3 and 4 of sample population, respectively, are considered rare in light of the fact that they have not been previously reported in the study area. There was high prevalence of the Y184F as well as the NF haplotype, having been detected in 84.6% of clinical isolates analysed. Consistent with this finding, the NF haplotype has been previously identified as the most commonly reported haplotype in Africa [20].

### Evaluation of the number of mutations detected in clinical isolates from each patient sample

**Fig. 1:**
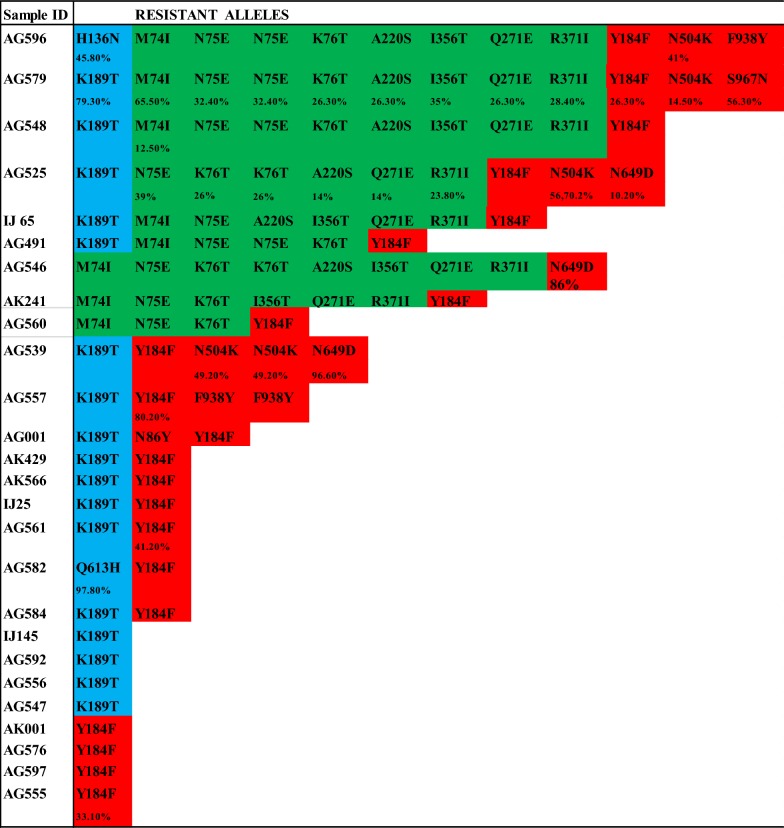
The individual sample data indicating the mutations detected by NGS in the *PfK13* (blue), *Pfcrt* (green), and *Pfmdr1* (red) genes. Variant frequency (VF) is the minimum fraction of reads that must contain a base for it to be called a variation (i.e. polymorphism). VF values of less than 100% are displayed with the specific mutation

Since there was a high preponderance of mutations found in the patient samples analysed, it was necessary to evaluate the number of mutations identified in each of the clinical isolates. Six of 26 (23%) of the sequenced isolates carried mutations in one or more codon position in each of the *Pfk13, Pfcrt* and *Pfmdr1* genes. Nineteen of the 26 (73.1%) isolates had one or more *Pfk13* mutations, 9 of the 26 (34.6%) isolates had one or more *Pfcrt* mutations, and 22 of the 26 (84.6%) isolates had one or more *Pfmdr1* mutations; see Fig. 1. The high prevalence of *Pfmdr1* mutations is largely attributed to the Y184F mutation, being found in 96% of the isolates that had mutations in this gene.

## Discussion

To proactively undertake surveillance for mutations that may lead to future resistance to ACT, sequencing data was generated from clinical isolates in individual patient samples in a high malaria endemic area using Sanger and NGS methods. Unlike other PCR based genotyping methods, which are only well suited for identification of major resistance alleles, the NGS method can accurately capture mixed infection haplotypes and detect minor resistance alleles as low as 1% [31, 32]. This study took advantage of the sensitivity of the NGS method in detecting the naturally circulating mutations in *P. falciparum* from individual patient samples. The results showed compelling evidence that the NGS method was more effective, detecting more mutations and haplotypes that were not identified by the Sanger method, thus, highlighting the efficacy of this method for surveillance of circulating anti-malarial resistance-associated alleles.

In 73.1% of the clinical isolates studied, *Pfk13* mutations consisting of two non-propeller mutations K189T and H136N and one propeller mutation Q613H were detected. Previously reported data from samples in this study area of Lagos, Nigeria showed no *k13* propeller mutations in the 89 samples evaluated by Sanger method [24]. Though the frequency of the propeller Q613H allele was low and its association with any resistance phenotype is unknown, its presence confirms the possibility of independent development of *k13* resistance genotypes as has been reported from other sites in Africa [8, 24, and 25]. Previous data from Africa has reported low levels of various *k13* propeller mutations, which is consistent with the findings of this research [24, 33]. There was a high prevalence (65.4%) involving a K189T mutation which has been previously associated with anti-malarial resistance, however, it is noteworthy that multiple mutations are required to functionally confer drug resistance [34].

Following reduction in chloroquine efficacy in the treatment of malaria and in line with the WHO recommendation, ACT was adopted and deployed as the first line drug in the therapy of malaria by the Nigeria malaria control programme in 2005 [35]; however, studies conducted years after chloroquine was withdrawn continued to show mutations related to chloroquine-resistance phenotypes in *Pfcrt* and *Pfmdr1* genes in parasite populations in Nigeria [36, 37]. Evidence abound that the occurrence of mutation is a continuous event, even after withdrawal of a drug. This is consistent with findings in Ethiopia which identified the *Pfcrt* K76T mutation, associated with chloroquine resistance, in 100% of their clinical isolates, even though chloroquine was withdrawn in Ethiopia in 1999 [38]. In contrast, significant decrease in *Pfcrt* mutations (K76T) associated with clinical resistance to chloroquine and increase in the wild type *Pfcrt* allele (K76) which correlated with reversal to chloroquine susceptibility has been reported in some African countries, many years after chloroquine withdrawal as a first-line treatment for malaria [39–41]. In addition, the K76T mutation identified in 34.6% of patient samples analysed has been previously associated with chloroquine and amodiaquine resistance and the triple mutant CVIET haplotype which was identified in 26.9% of the clinical isolates has been previously associated with chloroquine resistance [38]. The moderate prevalence of the K76T mutation and CVIET haplotype in this region is a significant finding which may suggest that selective pressure is still being evoked, possibly by previous indiscriminate chloroquine use, or occasional chloroquine use in the study area. Chloroquine use in indiscriminate self-medication in the treatment of presumed malaria is still adopted, partly because the drug is widely available and accessible [26].

The need for laboratory-based diagnosis of malaria cannot be overemphasized, considering the vagueness of symptoms. For example, a recent study conducted in the study area reported over diagnosis and overtreatment of malaria, giving ACT to children that were later confirmed negative for malaria [42]. Furthermore, the association of K76T with amodiaquine resistance could have important implications for the effectiveness of the ACT (artesunate/amodiaquine) which is currently being used as the alternate first line treatment for malaria in high endemic Nigeria.

The variability of strains circulating in a high malaria-endemic population, such as Nigeria, is not novel; however, it emphasizes the need for the molecular surveillance of these strains in the population, particularly those strains harbouring mutations with a previously demonstrated association with anti-malarial resistance.

The *Pfmdr1* N86Y and Y184F mutations which are reported in the clinical isolates studied are not associated with resistance to lumefantrine, compared with previous reports on wild-type haplotypes [8, 20, 43]. It has been observed that the N86 wild-type allele is more prevalent in recurrent infections following treatment with artemether/lumefantrine [18, 44], and this was confirmed by a recent meta-analysis of 31 clinical trials that revealed a five-fold risk of parasite recrudescence in patients infected with parasites expressing the wild-type N86 following artemether/lumefantrine treatment compared with those infected with N86Y mutant parasites [19]. Furthermore, the N86Y mutation has been shown to increase susceptibility to lumefantrine and while promoting resistance to chloroquine and amodiaquine [20], thus, the low prevalence of the N86Y mutation (7.7%) could imply that selective drug pressures are encouraging the preponderance of the wild-type phenotype to promote drug resistance. The effect of N86Y mutation on parasite response to chloroquine and amodiaquine is significant in parasites expressing the CVIET *Pfcrt* variant [20], thus, making the evaluation of regional associations between *Pfmdr1* and *Pfcrt* haplotypes important. Information on the geographic distribution of *Pfmdr1* and *Pfcrt* haplotypes and local selective drug pressures may help to inform decisions on selecting optimal ACT regimen.

High prevalence of the wild-type N86 allele (84.6%) in this study samples, that have been linked to decrease susceptibility to artemether/lumefantrine [19] portend negative implication for its future use. Currently, the selective pressure on artemisinin in Nigeria is low because its usage is still recent and access and usage of it as a frontline ACT is still limited [2]. Although it is unclear whether the clinical isolates in this study are directly resistant to any anti-malarial drugs, the high preponderance of mutations makes the association to resistance reasonable, and circulation of these mutations has the potential to culminate in treatment failure and repeat visits to the clinic. The argument is that, the increased use of ACT and preponderance of molecular markers linked to decrease sensitivity to its partner drug will increase the selective pressure for artemisinin resistance. Strikingly, some of the individual isolates carry mutations that cut across 2 or 3 of the resistance-associated genes, possibly allowing for the manifestation of multi-drug resistant parasites. Mutations in the *Pfk13, Pfcrt* and *Pfmdr1* genes were found in 6 of 26 (23%) of the sequenced isolates while mutations involving *Pfk13* and *Pfmdr1* genes were exhibited in 9 of 26 (34.6%) isolates, with amino acid substitutions in one or more codon in each of *Pfcrt* and *Pfmdr1* genes in 2 of 26 (7.7%) isolates.

Information on the preponderance of anti-malarial drug resistance-associated mutations is vital to understanding how resistance patterns emerge. As malaria control strategies intensify in the region and the country enters the pre-elimination phase, continued molecular surveillance using sensitive methods, such as the NGS, will be useful to track the evolution and prevalence of mutations in resistance associated genes. A study to correlate mutations detected with the clinical outcomes of treatment was not performed and this is a limitation to this study.

## Conclusion

The findings of rare mutations (N504K, N649D, F938Y and S967N) in genes associable with anti-malarial resistance in clinical isolates in this study, on the one hand, provide important insights into the continued evolving nature of the problems of malaria parasites when exposed to therapeutic interventions. On the other hand, in a country as Nigeria, with high endemicity of malaria, the emergence of mutant parasite may amount to a serious adverse public health consequence. Arguably, it is imperative that there is a valuable need for the adoption of sensitive molecular methods in malaria surveillance and control effort to ensure early detection of possible resistant strains to the highly recommended artemisinin-based combination therapy.

## Data Availability

The datasets used and/or analysed during the current study are available from the corresponding author on reasonable request
